# Renal glomerular and tubular responses to glutaraldehyde- polymerized human hemoglobin

**DOI:** 10.3389/fmed.2023.1158359

**Published:** 2023-06-13

**Authors:** Matthew C. Williams, Xiaoyuan Zhang, Jin Hyen Baek, Felice D’Agnillo

**Affiliations:** Laboratory of Biochemistry and Vascular Biology, Office of Blood Research and Review, Center for Biologics Evaluation and Research (CBER), U.S. Food and Drug Administration (FDA), Silver Spring, MD, United States

**Keywords:** hemoglobin oxygen therapeutics, heme oxygenase, tubular epithelium, glomerular podocyte, iron handling, endothelium, tubulointerstitial macrophage

## Abstract

Hemoglobin-based oxygen carriers (HBOCs) are being developed as oxygen and volume replacement therapeutics, however, their molecular and cellular effects on the vasculature and different organ systems are not fully defined. Using a guinea pig transfusion model, we examined the renal glomerular and tubular responses to PolyHeme, a highly characterized glutaraldehyde-polymerized human hemoglobin with low tetrameric hemoglobin content. PolyHeme-infused animals showed no major changes in glomerular histology or loss of specific markers of glomerular podocytes (Wilms tumor 1 protein, podocin, and podocalyxin) or endothelial cells (ETS-related gene and claudin-5) after 4, 24, and 72 h. Relative to sham controls, PolyHeme-infused animals also showed similar expression and subcellular distribution of N-cadherin and E-cadherin, two key epithelial junctional proteins of proximal and distal tubules, respectively. In terms of heme catabolism and iron-handling responses, PolyHeme induced a moderate but transient expression of heme oxygenase-1 in proximal tubular epithelium and tubulointerstitial macrophages that was accompanied by increased iron deposition in tubular epithelium. Contrary to previous findings with other modified or acellular hemoglobins, the present data show that PolyHeme does not disrupt the junctional integrity of the renal glomerulus and tubular epithelium, and triggers moderate activation of heme catabolic and iron sequestration systems likely as part of a renal adaptive response.

## Introduction

Chemically modified hemoglobin (Hb)-based oxygen carriers (HBOCs) are being developed as oxygen and volume replacement therapeutics for use as bridging agents when standard red blood cell (RBC) transfusions are not available, incompatible, or refused based on religious grounds (1, 2). HBOCs have also shown promise as oxygen-carrying perfusates in organ preservation settings (3, 4). Most HBOCs are manufactured from expired human or animal blood starting with the extraction and purification of Hb from RBCs, followed by various protein modification strategies designed to stabilize the acellular Hb in circulation and/or maintain its oxygen transport properties (2, 5, 6). Despite significant progress over many years, unresolved safety issues continue to impede the development and licensure of these therapeutics in the United States. These safety concerns have been driven, in part, by reports of adverse clinical events including transient hypertension, stroke, and myocardial infarction observed with some HBOCs (2, 7–9). Some studies have proposed that the clinical benefits of these products may still outweigh their safety risks, especially in severe anemia settings when RBCs are not an option (10–12). While there are no HBOCs licensed for human use in the United States, a glutaraldehyde-polymerized bovine Hb (Hemopure^®^, HbO_2_ Therapeutics, Souderton, PA, USA) was approved in South Africa for acute anemia settings (13).

Hemoglobin-based oxygen carrier-associated safety concerns have been difficult to resolve, partly because preclinical testing has not always been predictive of clinical safety outcomes for some HBOCs (7, 14). Moreover, there are important limitations related to the extrapolation of safety assessments in normal healthy animals to a heterogenous population of subjects with underlying comorbidities, endothelial dysfunction, and predisposition to various disease states (7, 14–16). This has prompted the search for preclinical approaches that may better predict HBOC safety in humans. Two areas of preclinical testing that have garnered some attention include the selection of relevant animal species and the use of sensitive biomarkers to help detect subtle safety signals (14, 17, 18). Previous studies from our laboratory indicate that the guinea pig may be a relevant small animal species to evaluate the unique pro-oxidative profiles of HBOCs (17, 19–23). This is based on the noted similarities between guinea pigs and humans in terms of their overall plasma and tissue antioxidant capabilities (14). Importantly, both species are unable to produce ascorbic acid (AA) due to an evolutionary loss of L-gluconolactone-oxidase, which is the rate limiting enzyme in AA synthesis. AA plays a key physiological role in limiting Hb oxidation, and thus the *in vivo* status of AA may be important in predicting the oxidation of HBOCs to methemoglobin and the effects of oxidized and/or degraded forms of Hb on tissues.

While the precise mechanisms underlying the reported adverse responses to HBOCs are not completely understood, the interaction of acellular Hb and/or its breakdown products with the vasculature of different organ systems is thought to be an important contributing factor (18, 24, 25). The kidney, for example, is recognized as being particularly susceptible to the adverse effects of high levels of circulating acellular Hb resulting from disease or drug-induced hemolysis, particularly when protective plasma protein defenses (e.g., haptoglobin) are overwhelmed (24, 26). This has been attributed to the glomerular filtration of dimerized Hb molecules, heme, and to a lesser extent native tetrameric Hb that can lead to acute tubular necrosis via oxidative and inflammatory mechanisms (26, 27). Renal failure and nephrotoxicity associated with early generation HBOCs were largely attributed to their high content of unmodified tetrameric Hb (28). Along these lines, we previously reported that chemically modified Hb and oxidized acellular Hb induced renal glomerular barrier dysfunction, downregulated the expression of key glomerular podocyte and endothelial intercellular junctional proteins, generated extensive renal iron deposition and oxidative stress in renal tubular epithelium, and suppressed the expression and activity of renal antioxidant enzymes (19, 21–23). Here, using a guinea pig transfusion model, we examine the renal glomerular and tubular responses to PolyHeme, a highly characterized glutaraldehyde-polymerized human Hb with low tetrameric Hb content. PolyHeme, manufactured by Northfield Laboratories (Evanston, IL, USA), was the subject of extensive clinical investigation but its production was discontinued following unsuccessful attempts to gain licensure in the United States (29, 30). Our study describes the renal responses to this highly characterized HBOC using a histological and immunohistological approach that may have useful implications for the preclinical assessment of other existing or emerging HBOC candidates.

## Materials and methods

### Polymerized Hb solution

PolyHeme is a chemically modified sterile Hb solution containing a heterogeneous mixture of glutaraldehyde-crosslinked and polymerized pyridoxylated human Hb at a concentration of 10 g/dl in an electrolyte solution. The product has a colloid oncotic pressure (COP) of 20–25 mmHg, a p50 of 26–32 mmHg, methemoglobin content of <5–8%, and an average molecular weight between 130 and 250 kDa with less than 1% tetramers. PolyHeme, originally manufactured by Northfield Laboratories (Evanston, IL, USA), was obtained via a Material Transfer Agreement from HbO_2_ Therapeutics (Souderton, PA, USA). Upon receipt, PolyHeme stocks were aliquoted under sterile conditions and stored frozen at −80°C. Prior to use, biochemical characterization experiments were performed to verify the quality and stability of existing PolyHeme stocks, including the assessment of total Hb concentration and oxidation state, aggregation, impurities, COP, and molecular size distribution using SDS-PAGE (5).

### Animal surgical preparation and experimental protocol

Male Hartley guinea pigs were purchased from Charles Rivers Laboratories (Wilmington, MA, USA) and acclimated for 1 week upon arrival at the FDA/CBER animal care facility. All animals were fed normal diets during the acclimation period and weighed 900–1,200 g at the time of the study. Animal study protocols were approved by the FDA/CBER Institutional Animal Care and Use Committee with all experimental procedures performed in accordance with the National Institutes of Health Guide for the Care and Use of Laboratory Animals (NIH Publications No. 8023, revised 1978). Surgical preparation and carotid catheter implantation were performed as previously described (17). Twenty-four hours after recovery from surgical catheter implantation, fully conscious and freely moving guinea pigs (*n* = 18 animals) underwent a 25% exchange transfusion (ET) (50 ml blood volume/kg body weight), replacing whole blood with PolyHeme at a rate of approximately 1 ml/min. Sham control animals underwent the surgical procedure and then recovered for 24 h. All animals were caged and freely moving up until the indicated necropsy time. To harvest the kidneys, sham control and PolyHeme-infused animals were euthanized by intraperitoneal injection of Euthasol^®^, femoral veins were cut, and cold saline was perfused via the arterial catheter to remove blood. Kidneys were dissected, cut in half, and fixed in 10% formalin for 24 h.

### Hb oxidation analysis

Spectral analysis of PolyHeme in plasma samples was performed using a rapid scanning diode array spectrophotometer (Model HP-8453, Agilent Technologies, Rockville, MD, USA). The concentration of total Hb and methemoglobin were determined using multi-component analysis (19).

### Histological analyses

Formalin-fixed paraffin-embedded (FFPE) kidney sections 3 μm thick were dewaxed in Safeclear II (xylene substitute), rehydrated in graded alcohol (100, 95, and 50%) and deionized water, and stained by standard hematoxylin-eosin (H&E) procedures. Whole slide brightfield imaging was performed using a Hamamatsu NanoZoomer 2.0-RS whole-slide digital scanner equipped with a 20× objective. Analysis software NDP.view2 was used for image processing (Hamamatsu Photonics, Japan). H&E-stained sections were used for glomerular morphometry measurements, including glomerular density and glomerular tuft area. For glomerular density, the number of glomeruli were counted irrespective of size in the outer-to-mid cortex regions of each kidney section. Glomerular density values were derived by dividing the number of glomeruli by the measured cortical area of the region (minimum total cortical area analyzed was 20–30 mm^2^ for each kidney section). Values for each animal per time interval were averaged and reported as the number of glomeruli per cortical area (*n* = 4–5 animals per group). For glomerular tuft area measurements, a total of 40–50 glomeruli per kidney were randomly selected from four different regions of the section. Tuft area values for each kidney section were measured using the NDP.view2 software and reported as the mean glomerular area in μm^2^ for each group.

### Immunofluorescence analyses

Formalin-fixed paraffin-embedded kidney sections 3 μm thick were dewaxed in Safeclear II (xylene substitute), rehydrated in graded alcohol (100, 95, and 50%) and deionized water, and heat-treated in a microwave oven for 15 min in 10 mM Tris/1 mM EDTA buffer (pH 9.0). After cooling for 30 min at room temperature (RT), heat-retrieved sections were blocked in phosphate-buffered saline containing 0.05% Tween-20 (PBST) and 2.5% bovine serum albumin (BSA) for 30 min at RT followed by overnight incubation at 4°C with primary antibodies in 1% BSA. Primary antibodies used included ETS-related gene (ERG, Biocare Medical, CM421), Wilms tumor protein (WT1, Abcam, ab89901), claudin-5 (CL5, Thermo Fisher Scientific, 35-2500), Iba1 (Abcam, ab5076), N-cadherin (NCAD, Cell Signaling, 13116S), E-cadherin (ECAD, Abcam, ab219332), podocin (Abcam, ab181143), podocalyxin (Abcam, ab203079), and heme oxygenase-1 (HO-1, Enzo Life Sciences, ADI-SPA-894). Sections were rinsed and incubated with Alexa Fluor 488 (A-21206) and Alexa Fluor 647-conjugated secondary antibodies (A-31571 and A-21447) for 1 h at RT (Thermo Fisher Scientific, Waltham, MA, USA). Nuclei were counterstained with Hoechst 33342. For double-labeling experiments, primary antibodies were mixed and incubated overnight at 4°C. For negative controls, sections were incubated without the primary antibody or mouse/rabbit isotype antibody controls. Sections stained with conjugated secondary antibodies alone showed no specific staining. Whole slide fluorescence imaging was performed using a Hamamatsu NanoZoomer 2.0-RS whole-slide digital scanner equipped with a 20× objective and a fluorescence module (#L11600). NDP.view2 software was used for image processing. Immunofluorescence and differential interference images were also captured using an Axio Observer Z1 inverted microscope (Carl Zeiss, Thornwood, NY, USA) equipped with an Axiocam 506 monochrome camera, an ApoTome.2 optical sectioning system, and a Plan-Apochromat 63×/1.4 NA oil immersion with WD = 0.19 and Plan-Apochromat 20×/0.8 objective lens. Digital image post-processing and analysis were performed using the ZEN 2 ver. 2.0 imaging software. Images were constructed from Z-stack slices collected at 0.48 μm intervals (5 μm thickness in total) and visualized as maximum intensity projections in orthogonal mode.

For WT1 or ERG semiquantitative analysis of podocytes or endothelial cells, respectively, a total of 20 glomeruli from randomly selected cortical areas per kidney section were acquired at 630× magnification and processed by Z-stack analysis using the ZEN 2 software. For each glomerulus, WT1 or ERG-positive nuclei were counted and divided by the glomerular tuft area. WT1 or ERG staining values for each section were averaged to derive a final score for each animal. The values were averaged to derive final scores for each group (*n* = 4 animals per group). For podocin, podocalyxin, and claudin-5 signal quantitation, relative fluorescence intensity values for a minimum of 10–15 randomly selected glomeruli per section were measured using the ZEN software. Average values per group were reported for each marker protein (*n* = 4 animals per group).

For semiquantitative analysis of proximal tubular HO-1 expression, a total of 40–50 randomly selected proximal tubular segments per kidney section were acquired at 630× magnification and processed by Z-stack analysis. Relative fluorescence intensity unit (RFU) values for each tubular segment were measured using the ZEN software, and average RFU values per group were calculated (*n* = 4 animals per group). Semiquantitative analysis of tubulointerstitial HO-1 expression was performed on whole-slide digital images of randomly selected cortical regions of each kidney section using the NDP.view2 software (6–10 images per section). For each image, threshold settings were applied to delineate HO-1-positive cells in the tubulointerstitium using ImageJ software ver.1.46 (National Institutes of Health, Bethesda, MD, USA). The percentage of HO-1-positive area for each image was measured, average values were derived for each section, and the mean percentage of HO-1 positive areas were calculated for each group (*n* = 4–6 animals per group).

### Iron histochemistry

Ferric iron deposition primarily associated with hemosiderin was detected using the Perls method with diaminobenzidine (DAB) intensification as previously reported (19, 31). Semiquantitative analysis of Perls-DAB staining was performed on high resolution whole-slide digital images of each kidney section using the NDP.view2 software. For each kidney, the entire cortex region was divided into four areas and staining for each area was graded on the following scale: 0, no detectable brown staining; 1, detectable light staining in scattered cortical tubules; 2, brown deposits detected in <50% cortical tubules; and 3, brown deposits detected in >50% cortical tubules. The values were averaged to derive the staining score per animal and the final score for each time point was derived for all the animals combined (*n* = 5–6 animals per group).

### TUNEL assay

Kidney sections were deparaffinized, hydrated, and pretreated with Proteinase K followed by EDTA, washed with distilled H_2_O, and blocked with 2.5% BSA. Sections were then incubated in a reaction mixture (TdT, dUTP, and buffer), washed, and incubated with anti-digoxigenin antibody. Sections were then visualized with alkaline phosphatase-ImmPACT Vector Red and counterstained with hematoxylin.

### Statistical analyses

Data values are expressed as mean ± SEM. Statistical comparisons were performed by a one-way ANOVA for planned comparisons and *post-hoc* Bonferroni’s test for multiple comparisons between equal size groups or Dunnett’s post-test when group comparisons involved unequal sizes (GraphPad Prism 5 software, La Jolla, CA, USA). In all analyses, *p* < 0.05 was taken as the level of statistical significance.

## Results

### Renal exposure to PolyHeme does not disrupt glomerular barrier integrity

**FIGURE 1:**
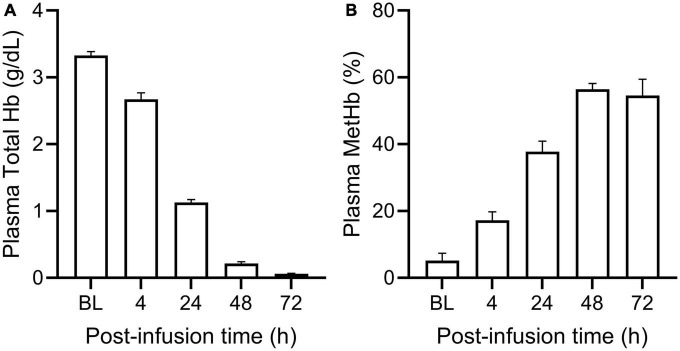
Total plasma Hb and methemoglobin levels in PolyHeme-infused guinea pigs. After a 25% exchange transfusion with PolyHeme (*n* = 18 total transfused animals), plasma samples were analyzed by spectrophotometry for the concentration of **(A)** total Hb and **(B)** methemoglobin. Serial samples were collected immediately after completion of infusion (baseline, BL) and at 4, 24, 48, and 72 h post-infusion.

PolyHeme administered as a 25% ET in guinea pigs produced a baseline or end-infusion maximum plasma Hb concentration of 3.3 ± 0.2 g/dl (*n* = 18 transfused animals) (Figure 1A). About 35% of this baseline concentration was still present in the circulation after 24 h but minimal levels remained 48 and 72-h post-infusion. In terms of the oxidative stability of PolyHeme, plasma methemoglobin levels rose from 5.2% at baseline to 17.2 and 37.8% at 4 and 24-h post-infusion, respectively (Figure 1B). Plasma methemoglobin measurements at 48 and 72-h are not considered meaningful given the minimal levels of circulating PolyHeme at these time points. Based on these initial data, renal tissues were collected at 4, 24, and 72 h post-infusion to investigate early, as well as late or persistent effects of PolyHeme on the kidney.

**FIGURE 2:**
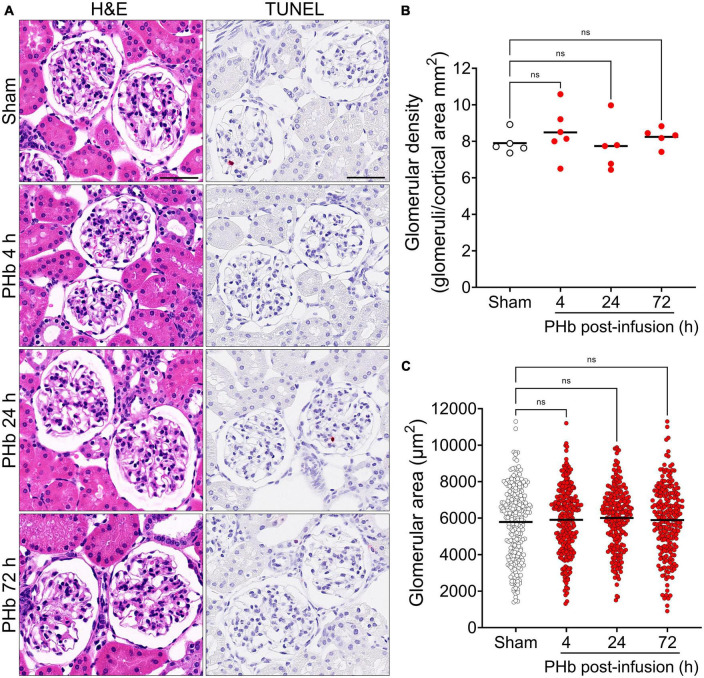
Glomerular histology and morphometric assessment in PolyHeme-infused guinea pigs. **(A)** Representative H&E and TUNEL staining of kidney sections from sham control animals or PolyHeme (PHb)-infused animals after 4, 24, and 72 h. Scale bars, 50 μm. **(B,C)** Measurement of glomerular density and glomerular tuft area using H&E-stained kidney sections from sham controls and PolyHeme-infused animals. **(B)** Glomerular density was determined by calculating the number of glomeruli per total renal cortical area (*n* = 5–6 animals per group). Glomerular density values for each animal and the means (black line) for each group are shown (*n* = 5–6 animals per group). **(C)** Glomerular tuft area was measured for 40–50 randomly selected glomeruli for each animal (*n* = 5–6 animals per group). The dots represent all the individual glomerular areas for each section and the means per group are shown (black line). ns, no statistical significance.

First, we sought to examine the effects of PolyHeme on the morphology and structural integrity of the renal glomerulus, as previous studies have reported that the glomerular filtration barrier is susceptible to Hb-induced damage (21, 23, 32). Routine H&E examination revealed no changes in the overall histology of glomeruli between sham controls or PolyHeme animals at 4, 24, and 72 h post-infusion (Figure 2A). TUNEL analyses also showed no major glomerular cell death following PolyHeme infusion (Figure 2A). Morphometric analyses showed no differences in glomerular density or average glomerular tuft area between sham controls and PolyHeme animals (Figures 2B, C).

**FIGURE 3:**
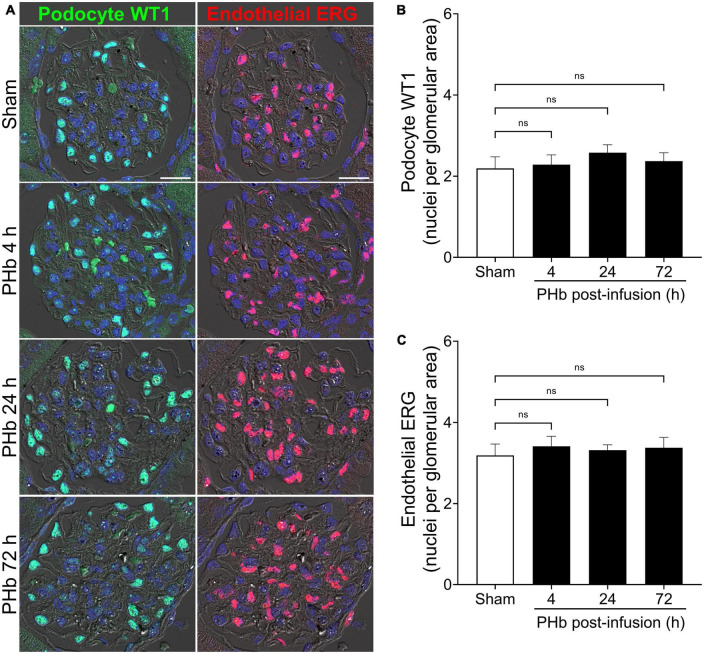
PolyHeme does not alter glomerular podocyte and endothelial density. **(A)** Representative immunofluorescence images combined with differential interference contrast of podocyte-expressed WT1 and endothelial-expressed ERG in serial kidney sections from sham control animals or PolyHeme (PHb)-infused animals after 4, 24, and 72 h. Nuclei were counterstained with Hoechst 33342 (blue). Scale bars, 20 μm. **(B,C)** Semiquantitative image analyses of podocyte WT1 and endothelial ERG. For each kidney section, the glomerular WT1 or ERG density values were derived by dividing the number of WT1 or ERG positive nuclei in each glomerulus by the glomerular tuft area (*n* = 15–20 randomly selected glomeruli per animal). The mean glomerular density values for each group are shown (*n* = 4 animals per group). ns, no statistical significance.

Next, we examined the effect of PolyHeme on glomerular podocytes and endothelial cells that together form the glomerular filtration barrier. To specifically identify these glomerular cell types, we analyzed the expression of podocyte WT1 and endothelial ERG by immunofluorescence microscopy (33, 34). Podocytes and endothelial cells showed intense and distinct nuclear localization of WT1 and ERG, respectively, consistent with their role as key transcription factors in these cells (Figure 3A). Semiquantitative image analyses revealed no significant change in the number of WT1-positive podocytes (Figure 3B) or ERG-positive endothelial cells (Figure 3C) between sham controls and PolyHeme animals at 4, 24, or 72 h post-infusion.

**FIGURE 4:**
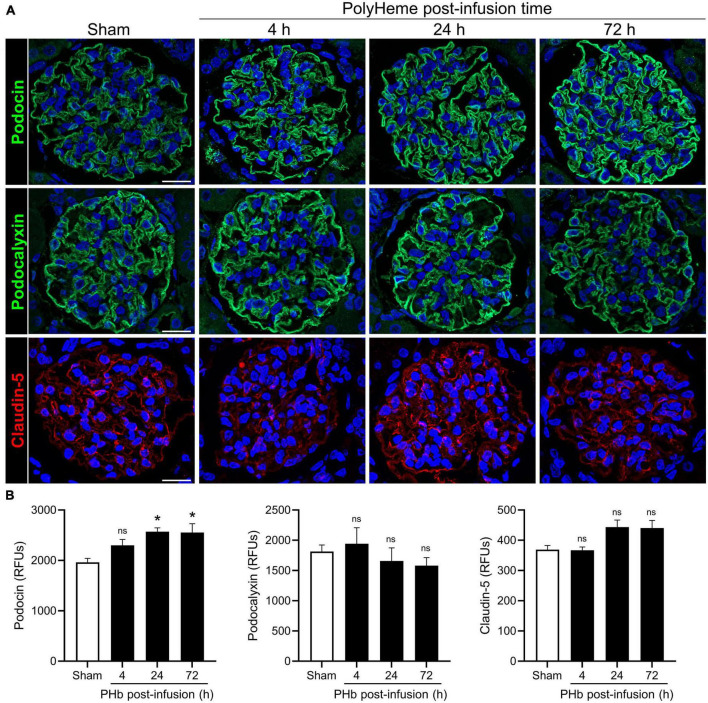
Effect of PolyHeme on podocyte and endothelial junctional and glycocalyx protein markers. **(A)** Representative immunofluorescence images of podocyte-expressed podocin and podocalyxin and endothelial-expressed claudin-5 in kidney sections from sham control animals or PolyHeme (PHb)-infused animals after 4, 24, and 72 h. Nuclei were counterstained with Hoechst 33342 (blue). Scale bars, 20 μm. **(B)** Semiquantitative image analyses of podocin, podocalyxin, and claudin-5. For each kidney section, the relative fluorescence intensity units (RFUs) of each respective protein were measured in 15–20 randomly selected glomeruli and the mean RFU values for each group were calculated (*n* = 4 animals per group). *p ≤ 0.05; ns, no statistical significance.

We then analyzed the expression of podocin (a main constituent of slit diaphragm complexes between podocyte foot processes), podocalyxin (a major podocyte sialoglycoprotein that readily binds and stabilizes components of the glomerular glycocalyx), and claudin-5 (a key component of endothelial tight junctions) (34, 35). Figure 4A shows the linear and uniform staining pattern of podocin and podocalyxin along podocyte structures compared to the linear but more diffuse staining of claudin-5 in the glomerular capillaries. Semiquantitative analyses of podocalyxin and claudin-5 immunofluorescence showed no significant changes in expression of these proteins over the course of 72 h post-PolyHeme infusion (Figure 4B). In the case of podocin, there was an increase in relative staining intensity that reached statistical significance at 24 and 72 h (Figure 4B). In contrast to previous studies that reported that exposure to other types of acellular Hb alters the structural integrity of the glomerular filtration barrier, the present findings indicate that PolyHeme does not induce a loss of glomerular podocytes or endothelial cells or downregulate the expression of key proteins involved in barrier function regulation.

### Effect of PolyHeme on the junctional integrity of renal tubular epithelium

**FIGURE 5:**
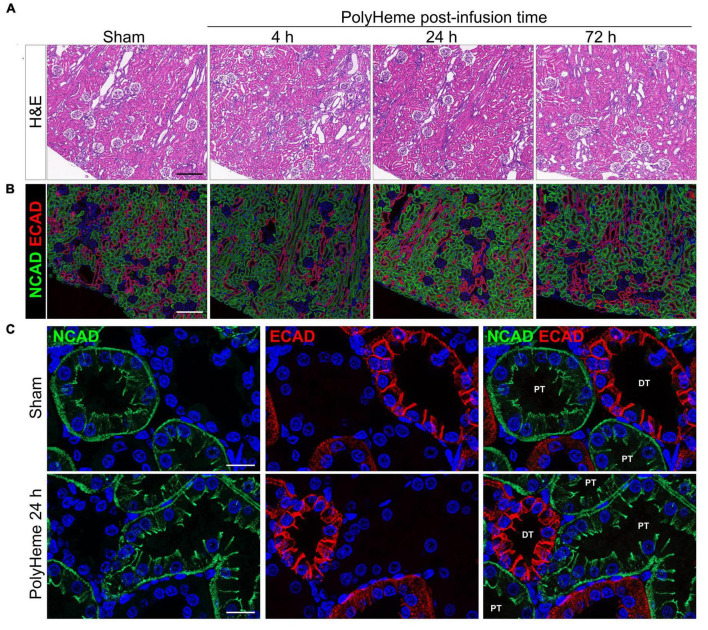
Effect of PolyHeme on epithelial junctional integrity of proximal and distal tubules. **(A,B)** Representative H&E and double-labeled immunofluorescence images of N-cadherin and E-cadherin in kidney sections from sham controls or PolyHeme-infused animals over the course of 72 h. **(C)** High resolution confocal imaging of the subcellular localization pattern of N-cadherin and E-cadherin in proximal and distal tubules, respectively. Scale bars, 250 μm **(A,B)**; 20 μm **(C)**. PT, proximal tubule; DT, distal tubule.

Next, we examined the effect of PolyHeme on the renal tubular epithelium, which is a common site of damage in heme or iron overload settings and is also involved in mounting protective responses against these insults (24, 26). H&E staining of renal sections from PolyHeme-infused animals revealed minimal tubular brush border loss, tubular dilation, or sloughing of cellular debris in cortical nephron segments over the course of 72 h (Figure 5A). To investigate the possibility that PolyHeme induced more subtle changes to intercellular junctional complexes of the tubular epithelium, we analyzed the expression of N-cadherin and E-cadherin, two key epithelial transmembrane proteins that are highly expressed in proximal and distal tubules, respectively. Immunofluorescence analyses revealed minimal changes in the overall tubular expression of both N-cadherin and E-cadherin between PolyHeme-infused animals and sham controls (Figure 5B). N-cadherin and E-cadherin immunolabeling was not detected in glomeruli or the vasculature. High resolution confocal imaging showed that N-cadherin in proximal tubules was localized diffusely along the basolateral cell border and as thin, concentrated intercellular bands near the apical cell lining, while E-cadherin in distal tubules was mainly expressed at intercellular cell contacts (Figure 5C). No major differences were observed in the subcellular distribution patterns of these proteins between PolyHeme-infused and sham control animals (Figure 5C).

### Renal HO-1 expression and iron deposition in PolyHeme-infused guinea pigs

**FIGURE 6:**
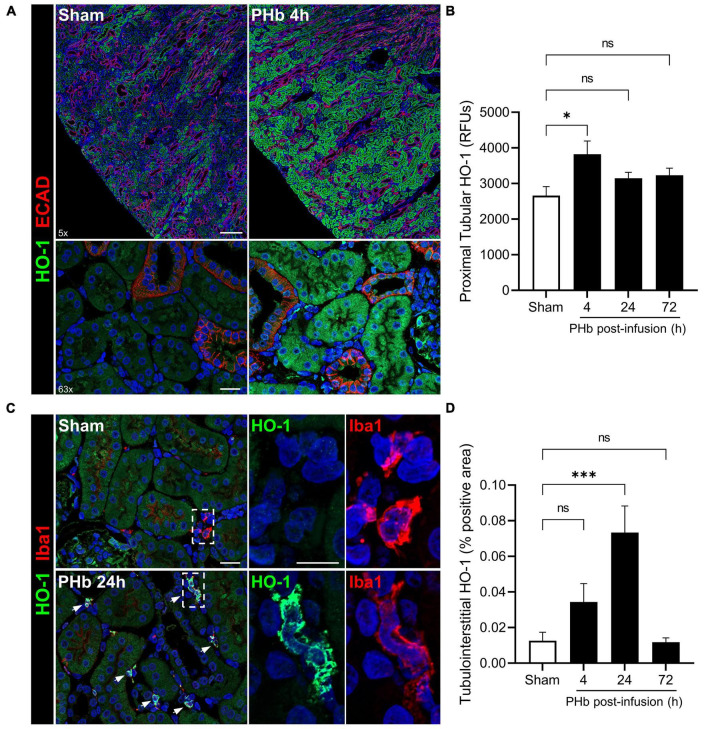
PolyHeme induces HO-1 in proximal tubular epithelium and tubulointerstitial macrophages. **(A)** Representative low and high magnification images of immunofluorescence staining for HO-1 and E-cadherin in cortical renal sections from sham controls or PolyHeme (PHb)-infused animals 4 h post-infusion. **(B)** Semiquantitative image analysis of HO-1 immunofluorescence intensity in proximal tubule segments. For each renal section, the RFU values of 40–50 randomly outlined proximal tubule profiles were measured, averaged, and the mean RFU values for each group were calculated (*n* = 4–5 animals per group). **(C)** Dual staining for HO-1 and macrophage marker Iba1 in sham controls or PHb-infused animals 24 h post-infusion. White arrows depict colocalized expression of HO-1 in Iba1-positive tubulointerstitial macrophages. **(D)** Semiquantitative analysis of tubulointerstitial areas positively labeled with HO-1-expressing tubulointerstitial macrophages. The mean area values for each group are shown (*n* = 4–5 animals per group). Scale bars, 250 μm (5× **A**); 20 μm (63× **A**); 20 μm **(C)**. *p ≤ 0.05; ^***^p ≤ 0.001; ns, no statistical significance.

Heme catabolism and iron sequestration systems play a critical protective role in the kidney and are highly activated by filtered heme, iron-bound transport proteins, or hemoprotein exposure (19, 24, 36, 37). To further evaluate the renal response to PolyHeme, we examined the expression of HO-1, an inducible rate-limiting enzyme of heme catabolism. Double-labeling experiments for HO-1 and E-cadherin expressed in distal tubules revealed increased HO-1 staining localized to cortical proximal tubular epithelium in PolyHeme-infused animals (Figure 6A). Semiquantitative image analysis of HO-1 expressed in proximal tubules confirmed a moderate but significant induction at 4 h but not at 24 and 72 h post-infusion (Figure 6B). No changes in glomerular HO-1 expression were detected between sham controls or PolyHeme-infused animals at any time interval. Further examination of these renal sections identified HO-1-positive cells in the cortical tubulointerstitium at 24 h post-infusion (Figure 6C). These HO-1 positive cells were identified as tubulointerstitial macrophages based on colocalization with macrophage marker Iba1. In sham controls, resident Iba1 positive tubulointerstitial macrophages showed no HO-1 expression (Figure 6C). Semiquantitative analyses revealed a significant increase in HO-1-positive cells in the cortical tubulointerstitium at 24 h, but not at 4 or 72 h post-infusion (Figure 6D).

**FIGURE 7:**
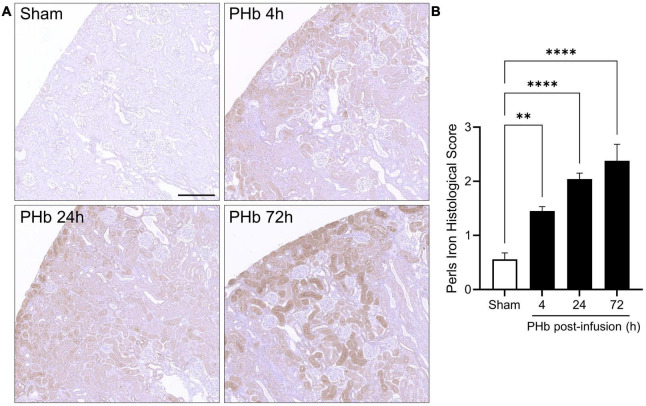
Renal iron deposition in PolyHeme-infused guinea pigs. **(A)** Non-heme iron staining using the Perls-DAB assay in sham control animals or at 4, 24, and 72 h after PolyHeme infusion. Perls-DAB reactivity (brown staining) is mainly observed in proximal tubules in PolyHeme-infused animals but not in glomeruli. Scale bars, 250 μm **(A)**. **(B)** Histological scoring of Perls iron deposition in sham control and PolyHeme-infused animals (*n* = 4–6 animals per group). ^**^p ≤ 0.005; ^****^p ≤ 0.0001.

Heme oxygenase-1 upregulation is often accompanied by the accumulation of ferritin and hemosiderin (a degradation product of excess ferritin stores) that helps trap iron generated by heme catabolism. To assess iron deposition, renal sections were stained using an enhanced Perls assay that mainly detects ferric iron in hemosiderin deposits. In the PolyHeme group, Perls-reactive iron staining was detectable in proximal tubule segments, but not in the glomeruli, at every time point post-infusion (Figure 7A). Histological scoring identified significant increases in Perls-reactive iron staining at 4, 24, and 72 h post-infusion (Figure 7B). Together, these HO-1 and iron deposition observations suggest that exposure to PolyHeme and/or its breakdown products may be sufficient to trigger an adaptive response in the renal tubular and tubulointerstitial compartments.

## Discussion

Our previous studies proposed the guinea pig as a physiologically relevant small animal species to evaluate the safety and efficacy of HBOCs, given the noted similarities in the overall plasma and tissue antioxidant profiles between guinea pigs and humans (17, 19, 22, 23). Many of these earlier studies were limited to the investigation of one well-characterized glutaraldehyde-polymerized bovine hemoglobin (Oxyglobin^®^, HbO_2_ Therapeutics, Souderton, PA, USA) that was made available to our group at the time. In this study, we evaluated PolyHeme to further explore how the properties of different HBOCs may impact renal glomerular and tubular responses in this animal model. Although PolyHeme development has long been discontinued, there may be value in studying this highly characterized HBOC in the context of identifying different preclinical approaches and as a surrogate model for other similar types of products.

The present study shows that PolyHeme undergoes significant oxidation to methemoglobin in the circulation of guinea pigs. Uncontrolled oxidation of Hb in the circulation can be detrimental to the effectiveness of HBOCs because methemoglobin and other oxidized species no longer carry oxygen and have a greater propensity to release toxic heme or free iron into the circulation (14, 25). Plasma and tissue antioxidant systems play a critical role in controlling the oxidation and breakdown of HBOCs; and thus, the selection of animal species with similar antioxidant profiles to humans is important to accurately assess methemoglobin formation in preclinical studies. As evidence of this point, we previously showed that a glutaraldehyde-polymerized bovine Hb oxidized more readily in the circulation of guinea pigs (a non-ascorbate-producing species), compared to rats (an ascorbate-producing species with plasma and tissue antioxidant capacities different than that of humans) (17). The reductive capacity of ascorbate has also been used to mitigate methemoglobin formation in clinical settings with HBOCs such as Hemopure^®^ (HbO_2_ Therapeutics, Souderton, PA, USA) (13). Studying animal species that can accurately recapitulate the redox activity of HBOCs *in vivo* may also be important for the proper assessment of Hb-mediated nitric oxide (NO) scavenging reactions thought to underlie the reported hypertensive effects of HBOCs (14, 24, 25).

The present findings show that PolyHeme has minimal effects on the structural and junctional integrity of the renal glomerulus and the proximal/distal tubular epithelium in guinea pigs. These results differ from some of the previous renal findings we observed with the transfusion of other modified or acellular Hbs. For example, we previously reported that the infusion of glutaraldehyde-polymerized bovine Hb containing a high content of unmodified (<5%) and stabilized Hb tetramers (∼35%) induced a transient disruption of renal glomerular barrier integrity and function that coincided with the reduced expression of glomerular podocyte and endothelial proteins (23). Guinea pigs infused with nitrite-oxidized human Hb also showed increased tubular and glomerular injury (21). Compared to these other solutions, PolyHeme contains a lower content of tetrameric Hb (<1%), which may partly explain the differences in glomerular and tubular responses. High molecular weight polymers of PolyHeme are less prone to glomerular filtration, thus limiting exposure of the tubular lumen to the product. Monitoring glomerular and tubular integrity using sensitive and specific biomarkers may allow for the assessment of subtle changes in the kidney nephron that may not otherwise be captured by traditional renal histological analyses. Intercellular junctional integrity and communication among podocytes, endothelial cells, and epithelial cells forming the glomerular filtration barrier and tubular segments of the nephron are particularly sensitive to changes in the microenvironment of the nephron (38–42). The loss or abnormal localization of these junctional adhesion molecules characterizes the pathogenesis of various renal injury or disease states (38, 43, 44).

Heme oxygenase (HO), the rate-limiting enzyme of heme catabolism, exists as two main isoforms: inducible HO-1 and constitutive HO-2. HO catalyzes the degradation of heme to biliverdin, carbon monoxide, and ferrous iron, which is rapidly exported from the cell via ferroportin or sequestered by ferritin for storage. HO-1 is up-regulated by exposure to Hb, heme, lipopolysaccharide, and several other pro-oxidant stimuli (36). In this study, PolyHeme induced a moderate and transient induction of HO-1 in the renal proximal tubular system. These observations suggest that there may be some level of exposure of these compartments to PolyHeme and/or its breakdown products. While an intact glomerular barrier may prevent the passage of high molecular weight forms of PolyHeme, we suspect that the filtration and tubular uptake of smaller degraded heme (bound/unbound) or iron-containing species derived from intravascular product breakdown or extravascular catabolism may have been sufficient to trigger the tubular HO-1 response. The increased oxidation of PolyHeme to methemoglobin would lend support to this hypothesis, as oxidized Hb is more easily degraded and prone to liberate heme. Consistent with this idea, we previously reported that differences in methemoglobin formation between rats and guinea pigs infused with Oxyglobin may differentially modulate the activation of heme catabolic and iron sequestration systems (19). Our present findings also revealed increased HO-1 expression in Iba1-positive tubulointerstitial macrophages, implying the possible exposure and cellular uptake of degraded heme or iron-containing species and possibly also intact PolyHeme polymers escaping the more permeable peritubular capillaries. Both sham controls and PolyHeme animals showed a similar degree of tubulointerstitial Iba1 staining with no clear histological evidence of tubulointerstitial damage, suggesting that this HO-1 induction occurs mainly in resident tubulointerstitial macrophages and not in cellular infiltrates attracted to this site by inflammatory or injury signals. Macrophage HO-1 induction is also observed in organs such as the liver and spleen, which likely serve as more prominent sites of PolyHeme degradation and removal than the kidney (Supplementary Figure 1). Previous studies in chimpanzees and guinea pigs with similar glutaraldehyde polymerized Hbs reported a shift from renal excretion to reticuloendothelial elimination that correlated with the degree of Hb polymerization and/or the content of unmodified Hb (45, 46).

Many studies have provided evidence that HO-1 upregulation serves as an adaptive response that confers protection through anti-inflammatory and antioxidant mechanisms (36). HO-1 upregulation is often accompanied by the accumulation of ferritin and hemosiderin stores as a mechanism to trap liberated iron and limit toxicity (37). Conversely, under settings of excess heme exposure, HO-1 over-activation may promote iron or other byproduct toxicities (14, 27). In the case of this study, we speculate that the HO pathway may have provided some degree of protection to the kidney at these levels of PolyHeme exposure. It should also be noted that the iron deposition detected by the Perls method mainly represents the accumulation of hemosiderin, an iron storage complex with minimal free radical-generating potential. Thus, given the absence of renal damage in this study, the observed iron deposition may be interpreted as an indicator of exposure to PolyHeme or its degradation products rather than a marker of toxicity.

Protein modification strategies based on Hb polymerization have afforded nephroprotective benefits over early unmodified or intramolecularly cross-linked HBOC candidates (1, 28, 47). Although there is a lack of detailed published information on the renal toxicology of PolyHeme in animals, clinical studies have reported that PolyHeme does not cause overt renal toxicity (29, 30). There remains an important need to better understand and predict the renal effects of HBOCs in recipients that may be affected by pre-existing conditions including advanced age, diabetes, infection, ischemia, and chronic disease that render kidneys more susceptible to Hb or heme toxicity (26, 48–50). In this regard, the examination of HBOCs in relevant animal models of early or advanced endothelial dysfunction, inflammation, renal impairment, and traumatic shock should provide important information on safety and efficacy with potential implications for clinical trial design (14, 51).

## Conclusion

The present study shows that the infusion of PolyHeme produces minimal damage to the junctional integrity of the renal glomerulus and tubular epithelium in this guinea pig model. The observed moderate activation of heme catabolic pathways and non-heme iron deposition likely reflects the induction of a renal protective response. Reported similarities in the clinical adverse event profiles of different HBOC formulations have led to suggestions of common mechanisms of toxicity with this class of products. However, it is important to recognize that not every HBOC is created equally. Differences in key physiochemical properties related to oxygen affinity, redox activity and stability, molecular size and shape, and extravasation potential can profoundly influence preclinical and clinical responses to these products (1, 5, 47). From a preclinical perspective, the application of relevant small animal models and sensitive biomarkers that take into consideration the unique pro-oxidative properties of HBOCs may provide important comparative information in future investigations of existing or emerging HBOC candidates.

## Data availability statement

The raw data supporting the conclusions of this article will be made available by the authors, without undue reservation.

## Ethics statement

The animal study was reviewed and approved by the FDA/CBER Institutional Animal Care and Use Committee.

## Author contributions

FD’A conceived and designed the study, performed the statistical analysis, and wrote the first draft of the manuscript. FD’A, JHB, MCW, and XZ performed the animal studies and experimental analyses. XZ organized the animal database. FD’A and MCW revised the manuscript. All authors read and approved the submitted version.
