# Supplementary material for: Renal glomerular and tubular responses to glutaraldehyde- polymerized human hemoglobin
Source: Front Med (Lausanne). 2023 Jun 13;10:1158359. doi: 10.3389/fmed.2023.1158359 (PMC10293615; doi:10.3389/fmed.2023.1158359)
Supplement: Supplementary file 1 [file Image_1.PDF]

Supplementary Figure 1

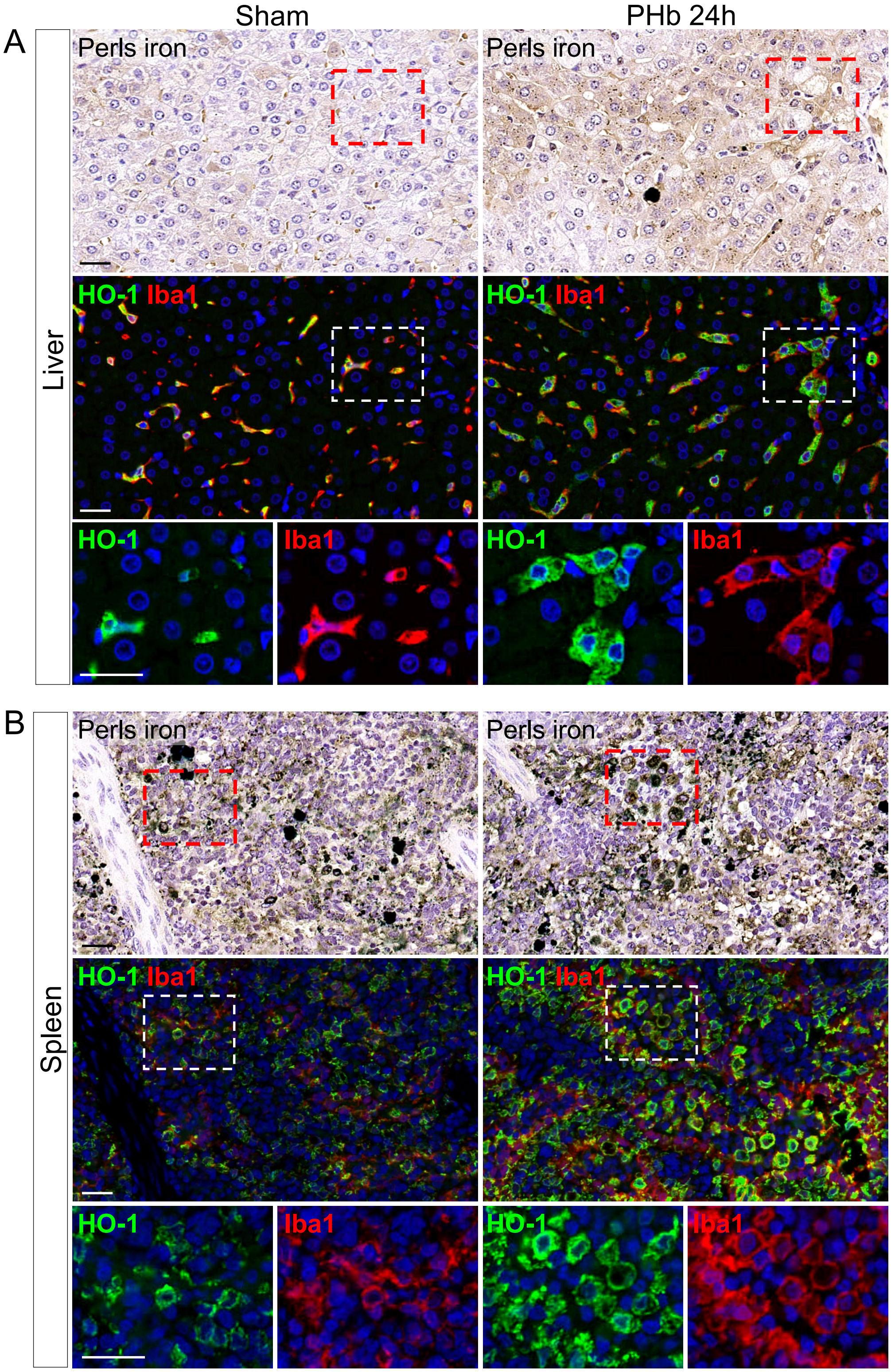

Supplementary Figure 1. HO-1 induction and iron deposition in the liver and spleen following PolyHeme infusion. Serial sections of (**A**) liver and (**B**) spleen from sham controls or PolyHeme (PHb)-infused animals 24 hours post-infusion were stained for non-heme iron deposition (Perls-DAB assay) and immunostained for HO-1 and Iba-1 (macrophage marker). In PHb-infused animals, Iba-1-positive macrophages in the liver and spleen show increased HO-1 expression and iron staining compared to sham controls. White boxes depict areas of magnification with colocalized expression of HO-1 in Iba1-positive macrophages, and the red boxes highlight the Perls iron staining in these identical regions. Nuclei were counterstained with Hoechst 33342 (blue). Scale bars = 25 μm.
